# Treatment Outcomes in Tuberculosis Patients with Diabetes: A Polytomous Analysis Using Brazilian Surveillance System

**DOI:** 10.1371/journal.pone.0100082

**Published:** 2014-07-08

**Authors:** Bárbara Reis-Santos, Teresa Gomes, Rodrigo Locatelli, Elizabete R. de Oliveira, Mauro N. Sanchez, Bernardo L. Horta, Lee W. Riley, Ethel L. Maciel

**Affiliations:** 1 Lab-Epi UFES Laboratory of Epidemiology of Universidade Federal do Espírito Santo, Vitória, Espírito Santo, Brazil; 2 Post-Graduate Programme in Epidemiology, Universidade Federal de Pelotas, Rio Grande do Sul, Brazil; 3 Post-Graduate Programme in Saúde Coletiva, Centro de Ciências da Saúde, Universidade Federal do Espírito Santo, Vitória, Espírito Santo, Brazil; 4 Department of Public Health, Faculdade de Ciências da Saúde, Universidade de Brasília, Brasília, Brazil; 5 Division of Infectious Disease and Vaccinology, School of Public Health, University of California, Berkeley, California, United States of America; Brighton and Sussex Medical School, United Kingdom

## Abstract

**Background:**

The impact of non-communicable diseases on tuberculosis incidence has received significant attention. It has been suggested that the risk of tuberculosis is higher among subjects with diabetes and these subjects also has poor TB treatment outcomes.This study was aimed at assessing the socio-demographic and clinical factors that may influence different outcome of TB in patients with DM (TB-DM) identified in the Brazilian national database from 2001 to 2011.

**Methods:**

TB-DM cases reported in the Brazilian information system were identified and compared.Covariates associated with the outcomes of interest (cure, default, deaths, and development of TB MDR) were included in a hierarchical regression model.

**Results:**

TB-DM cases increased from 380/100,000/year in 2001 to 6,150/100,000/year in 2011. Some of the main associations found are pointed. The odds of default was higher among those in the age group 20–39 years (OR = 2.07, 95%CI 1.32–3.24); alcoholics (OR = 2.17, 95%CI 1.86–2.54), and HIV/AIDS (OR = 2.16, 95%CI 1.70–2.74);positive monitoring smear (OR = 1.94, 95%CI 1.55–2.43); prior default (OR = 5.41, 95%CI 4.47–6.54), and unknown type of treatment (OR = 3.33, 95%CI 1.54–7.22). The odds of death was greater for subjects ≥60 years old (OR = 2.74, 95%CI 1.74–4.29); institutionalized in shelter (OR = 2.69, 95%CI 1.07–6.77); alcoholics (OR = 2.70, 95%CI 2.27–3.22); HIV/AIDS (OR = 2.87, 95%CI 2.13–3.86); pulmonary+extrapulmonary TB (OR = 2.49, 95%CI 1.79–3.46); with unknown type of treatment (OR = 14.12, 95%CI 7.04–28.32).Development of MDR TB was more related to relapse (OR = 9.60, 95%CI 6.07–15.14);previous default (OR = 17.13, 95%CI 9.58–30.63); and transfer of treatment center (OR = 7.87, 95%CI 4.74–13.07).

**Conclusions:**

Older subjects and those with comorbidities and with a previous treatment of TB had poorest outcomes. TB control program in Brazil will need to expand efforts to focus on treatment of TB-DM patients to improve their cure rates in order to achieve the goals of tuberculosis elimination.

## Introduction

Several health indicators point to the severity and importance of tuberculosis (TB) as a public health problem [1]. The clinical manifestation and treatment outcomes of TB is greatly influenced by its interaction with a variety of other factors, such as HIV infection, immunosuppressive chemotherapy, diabetes, as well as socio-demographic, socio-economic, and environmental factors [2].

Diabetes mellitus (DM) is a non-communicable chronic disease whose incidence is increasing globally [3]. The risk of TB development in subjects with DM is higher than that in the general population [4]. Besides that, once DM patients had TB, these subjects also could have an increased risk of poor TB treatment outcomes, including treatment failure, death, and relapse [4], [5]. In a recent paper that compared patients with TB and with and without DM in Brazil we found that those patients with TB and DM had poorer TB treatment outcome even after control to socio-demographic and clinical characteristics [6]. These results agree with previous studies published in different settings [7], [8].

Thus, to support the best care of patients with TB and DM and the implementation of targeted strategies by the control programs, it is important to know which characteristics that differentiated patients more likely to being cured in TB treatment of those with poor outcomes as default, death, or development of multidrug resistant TB (MDR TB). This study was aimed at assessing the socio-demographic and clinical factors that may influence different outcome of TB patient with DM identified in the Brazilian national database from 2001 to 2011.

### Study Population and Methods

SINAN is the Brazilian Information System for notifiable diseases that provides data accessible to the public via website maintained by the Data Processing Department of Brazilian Ministry of Health (DATASUS) [9], [10]. Despite some limitations inherent to any nationwide information system, SINAN is considered a fairly consistent and complete data source [11].

Cases of TB, diagnosed according to Brazilian guideline [12], reported to SINAN between 2001–2011 and that were classified as diabetics were identified and evaluated for socio-demographic characteristics, presence of comorbidities, as well as clinical features and TB treatment history. DM status at SINAN dataset is reported as dichotomous variable (no/yes) without information about the nature of DM development and is defined as presence of previous medical diagnosis of DM self-reported at TB notification.

The subjects were classified according to the following treatment outcome: cure, default, death from TB, death from other causes, and MDRTB.Those subjects whose TB treatment outcome was missing (transferred and missing data) were excluded of analysis.

The following socio-demographic variables were evaluated: age (0–19 years, 20–39 years, 40–59 years and ≥60 years), gender (male/female), skin color [white/black/browns/others (Indian/Asian)], schooling (illiterate/1–4 years/4–8 years/9–11 years/12–16 years/not applicable), area of residence (urban/rural or periurban) and whether the individual was institutionalized (no/prison/shelter/orphanage/psychiatric hospital/others – formal institutions did not include previously).

Regarding comorbidities, we assessed previous diagnosis of self-reported mental diseases (no/yes), alcoholism (no/yes), HIV/AIDS (negative/positive), and other comorbidities (no/yes). “Other comorbidities” is an open field of notification chart where healthcare worker should included any others diseases reported by the patients.

The covariates related to TB included: TB form (pulmonary/extra pulmonary/pulmonary + extrapulmonary); tuberculin skin test (negative/positive if higher than 10+ mm); X-ray suspicious for TB (no/yes); results of initial smear and second month smear (negative/positive), result of initial culture examination (negative/positive), and result of initial histopathologic examination [not suggestive of TB/suggestive/acid-fast bacilli (AFB) positive]; subjects under directly observed therapy [DOT (no/yes)]; and whether TB transmission was occupational [from the field “disease relates to work” (no/yes)].

To estimate the proportions of subjects with DM among TB cases, we divided the number of TB-DM subjects by the number of TB incident cases and multiplied by 100,000. We fitted a generalized linear model through the Poisson regression to analyze the trend of incident cases of TB and TB-DM where time (in years elapsed) was the independent variable [13]. Also, we estimated the percentage of variation according to the expression:
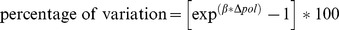



Where β is the regression coefficient and 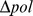
is the time series variation, in years [14].

Pearson chi-square testor likelihood-ratio chi-square testwas used to compare proportions. Covariates associated with the outcome of interest (p≤0.05) were included in a hierarchical regression model. In this analysis “cure” was the reference category for the outcome covariate and was compared with the others outcomes. Polytomous logistic regression is a useful technique for simultaneously modeling predicted probabilities of multiple outcome categories. The simultaneous consideration of multiple (differential) conditions serves clinical practice better than consideration of the presence of only one target condition as the outcome variable [15].

In the hierarchical model, the following covariates were included ranging from distal determinants to proximate categories [16]: level 1 (gender + age + schooling + skin color); level 2 (variables retained from level 1 + institutionalization + area of residence + occupational disease); level 3 (variables retained from level 2 + alcoholism + mental disease + others comorbidities + HIV/AIDS); level 4 (variables retained from level 3 + TB form + initial smear + smear 2nd month + X ray suspicious for TB + culture + histopathologic examination + TST); and level 5 (variables retained from level 4 + treatment type + DOT).

In each level, those covariates associated with the outcome (p≤0.10) were retained in the model. All analyses were performed with Stata, version 12.0.

The study was approved by the ethical committee of the Centro de Ciências da Saúde (Center of Health Sciences) of the Universidade Federal do Espírito Santo – number 242,831; March 10, 2013.

## Results

**Figure 1:**
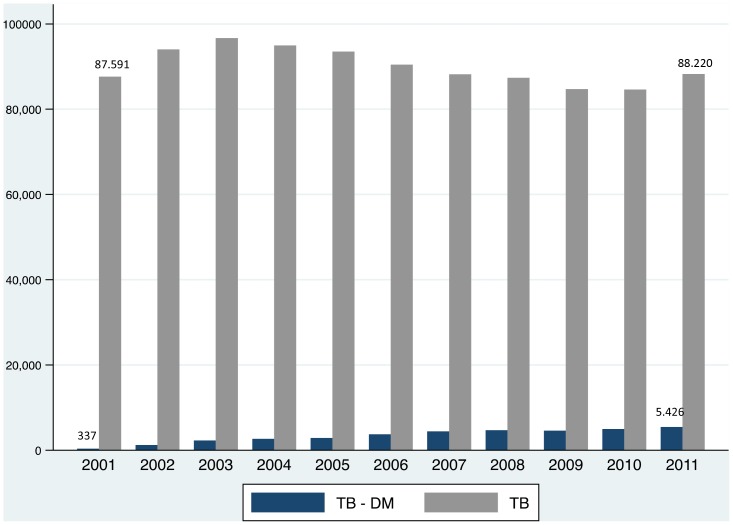
Distribution of Brazilian cases of tuberculosis and tuberculosis with diabetes notified at SINAN (Brazilian Information System for Notifiable Diseases), between 2001 and 2011.

Between 2001 and 2011, 990,017 cases of tuberculosis were reported to SINAN, and 36,920 (3,730/100,000) of these subjects were reported to have diabetes. However, the distribution of the cases was not uniform throughout this period. While the overall TB incident cases slightly decreased (RR = 0.990, 95% CI 0.990–0.991; variation 9.6%), the proportion of diabetics among TB cases increased progressively from 380/100,000/year in 2001 to 6,150/100,000/year in 2011 [RR = 1.162, 95% CI 1.158–1.166; variation 421% (Figure 1)], both statistically significant.

Because of missing information on TB treatment outcome, 10,519 were excluded. Therefore, 26,401 subjects were included in the present analyses, of which 21,110 (80%) were cured; 1,942 (7%) defaulted treatment, 1,128 (4%) died from TB, 2,082 (8%) died from other causes and 139 (1%) developed MDR TB.

Cure was slightly higher among females (82% vs78% in males, p<0.001) and was also more likely among subjects <20 years (87%). Default rate was 12% among those 20–39 years; was 6% for whites and 10% for blacks (p<0.001). Among those ≥60 years 13% died from causes other than TB (p<0.001).

Alcoholism accounted for 11% of TB deaths (p<0.001), while mental diseases and other comorbidities accounted for 13% (p<0.001) and 8% (p<0.001) of deaths from TB, respectively (Table 1).

**Table 1:** Distribution of socio-demographic characteristics of tuberculosis cases according to treatment outcome in Brazil—2001–2011.

Characteristics		Cure %	Default %	Death from TB %	Death from other cause %	MDRTB %	p value*
Gender	Female (10,072)	82	6	4	7	1	<0.001
	Male (16,329)	78	8	5	8	1	
Age	<20 years (430)	87	5	3	5	0	<0.001
	20–39 years (3,783)	81	12	2	4	1	
	40–59 years (13,726)	82	7	4	6	1	
	≥60 years (8,455)	75	5	6	13	0	
Skin color	White (9,964)	81	6	4	8	1	<0.001
	Black (3,004)	77	10	4	8	1	
	Brown (9,092)	80	8	4	7	1	
	Others (399)	84	8	2	6	0	
School level	Illiterate (2,365)	78	8	4	10	0	<0.001
	1–4 years (6,034)	80	8	4	7	1	
	5–8 years (4,968)	82	7	3	7	1	
	9–11 years (2,752)	85	7	3	4	1	
	12–16 years (1,205)	88	6	2	4	0	
	Not applicable (417)	83	4	5	8	0	
Area of residence	Urban(19,300)	80	8	4	7	1	0.005
	Rural (1,642)	79	6	4	10	1	
	Periurban(135)	81	5	2	12	0	
Institutionalization	No (13,072)	79	7	6	7	1	0.050**
	Penitentiary (346)	79	9	6	6	1	
	Shelter (37)	60	8	16	16	0	
	Orphanage (23)	91	4	0	5	0	
	Psychiatric hospital (28)	68	14	7	7	4	
	Others (406)	75	10	7	8	0	
Alcoholism	No (13,477)	81	6	5	7	1	<0.001
	Yes (2,174)	67	14	11	7	1	
Mental disease	No (14,866)	79	7	6	7	1	<0.001
	Yes (672)	73	13	6	7	0	
Others comorbidities	No (11,042)	81	7	5	6	1	<0.001
	Yes (3,066)	69	8	10	12	1	
HIV/AIDS	Negative (10,636)	84	6	4	5	1	<0.001
	Positive (831)	60	12	7	20	1	

MDR TB: multidrug resistant tuberculosis; TB: tuberculosis.

*Pearson chi-square test.

**Likelihood-ratio chi-square test.

Table 2 describes the study population according to form of TB. Among subjects with pulmonary plus extra pulmonary disease 10% died from TB and 16% died from others cause (p<0.001).

**Table 2:** Distribution of presentation and treatment characteristics of tuberculosis cases according to treatment outcome in Brazil—2001–2011.

Characteristics		Cure %	Default %	Death from TB %	Death from other cause %	MRTB %	p value*
TB form	Pulmonary (23,323)	81	7	4	7	1	<0.001
	Extrapulmonary (2,484)	74	7	5	15	0	
	Pulmonary+Extrapulmonary (593)	67	7	10	16	0	
Initial smear	Negative (5,366)	78	7	5	10	0	<0.001
	Positive (16,375)	83	7	3	6	1	
Tuberculin skin test	Negative (1,258)	77	7	4	12	0	<0.001
	Positive (2,975)	87	6	1	6	0	
Culture	Negative (1,377)	84	5	2	8	1	<0.001
	Positive (2,582)	81	7	3	6	3	
Histopathologic	Not suggestive (199)	71	9	4	14	2	0.001
	Suggestive (1,440)	81	6	3	10	0	
	AFB positive (1,156)	81	6	3	9	1	
X-ray suspicious for TB	No (1,181)	77	7	3	13	0	<0.001
	Yes (22,038)	80	7	4	8	1	
2^nd^ month smear	Negative (7,972)	93	3	1	3	0	<0.001
	Positive (2,083)	88	6	1	3	2	
DOT	No (7,344)	78	9	6	6	1	<0.001
	Yes (6,681)	84	5	4	6	1	
Occupational	No (16,496)	80	7	4	8	1	<0.001
	Yes (485)	86	6	3	4	1	
Treatment type	New case (22,712)	82	6	4	8	0	<0.001
	Relapse(1,555)	73	10	5	9	3	
	Return after default (746)	52	31	7	7	3	
	Unknown (86)	35	11	24	30	0	
	Transferred of treatment center (1,301)	78	7	5	8	2	

AFB: acid fast bacilli; DOT: directly observed therapy; MDR TB: multidrug resistant tuberculosis; TB: tuberculosis.

*Pearson chi-square test.

Among those subjects classified as new cases of TB, 82% were cured. On the other hand, among those who were classified as “unknown” at baseline only 35% were cured, 11% defaulted treatment, 24% died from TB and 30% died from other cause (p<0.001).

In the hierarchical polytomous regression model (Table 3), we observed that males had greater odds of default treatment (OR = 1.27, 95% CI 1.15–1.40), death from TB (OR = 1.37, 95% CI 1.20–1.56) and death from other causes (OR = 1.31, 95% CI 1.19–1.44), using cure as the reference group.

**Table 3:** Hierarchical* polytomous regression of the association of TB treatment outcome and tuberculosis subjects characteristics in Brazil—2009.

Characteristics			Cure-Default	Cure-Death from TB	Cure-Death from other cause	Cure-MRTB
			OR	OR	OR	OR
			(CI)	(CI)	(CI)	(CI)
Level 1	Gender	Female	Ref.	Ref.	Ref.	Ref.
		Male	1.27	1.37	1.31	1.02
			(1.15–1.40)	(1.20–1.56)	(1.19–1.44)	(0.72–1.44)
	Age	<20 years	Ref.	Ref.	Ref.	Ref.
		20–39 years	2.07	0.97	0.86	2.40
			(1.32–3.24)	(0.53–1.38)	(0.53–1.38)	(0.31–18.54)
		40–59 years	1.25	1.35	1.12	2.42
			(0.80–1.94)	(0.74–2.46)	(0.71–1.76)	(0.32–18.15)
		≥60 years	0.95	2.47	2.74	1.69
			(0.60–1.48)	(1.35–4.52)	(1.75–4.29)	(0.22–12.96)
	Skin color	White	Ref.	Ref.	Ref.	Ref.
		Black	1.52	1.25	1.06	1.49
			(1.31–1.76)	(1.02–1.54)	(0.91–1.23)	(0.90–2.47)
		Brown	1.19	1.26	0.90	1.26
			(1.06–1.33)	(1.09–1.45)	(0.81–1.01)	(0.86–1.84)
		Others	1.23	0.51	0.65	0.50
			(0.85–1.79)	(0.25–1.05)	(0.42–1.01)	(0.07–3.61)
	School level	Illiterate	Ref.	Ref.	Ref.	Ref.
		1–4 years	0.92	1.14	0.81	1.52
			(0.76–1.10)	(0.89–1.46)	(0.68–0.96)	(0.73–3.17)
		5–8years	0.77	0.92	0.80	1.90
			(0.64–0.93)	(0.70–1.20)	(0.66–0.95)	(0.91–3.98)
		9–11years	0.74	0.74	0.54	1.90
			(0.60–0.92)	(0.54–1.03)	(0.42–0.68)	(0.86–4.19)
		12–16years	0.58	0.55	0.45	0.40
			(0.43–0.78)	(0.34–0.88)	(0.32–0.62)	(0.08–1.88)
Level 2	Area of residence	Urban	Ref.	Ref.	Ref.	Ref.
		Rural	0.72	1.01	1.20	0.80
			(0.58–0.89)	(0.78–1.30)	(1.00–1.43)	(0.39–1.66)
		Periurban	0.61	0.53	1.55	5.11
			(0.28–1.32)	(0.17–1.69)	(0.91–2.66)	---
	Institutionalization	No	Ref.	Ref.	Ref.	Ref.
		Jail	1.00	1.05	0.98	0.78
			(0.68–1.47)	(0.66–1.65)	(0.62–1.54)	(0.19–3.22)
		Shelter	1.62	2.69	2.30	4.60
			(0.48–5.45)	(1.07–6.77)	(0.91–5.80)	---
		Orphanage	0.45	4.39	0.48	3.31
			(0.06–3.34)	---	(0.06–3.66)	---
		Psychiatric	1.73	1.20	1.23	5.00
		hospital	(0.58–5.16)	(0.27–5.23)	(0.28–5.38)	(0.65–38.40)
		Others	1.41	1.28	1.17	5.11
			(1.01–1.97)	(0.87–1.89)	(0.80–1.72)	---
	Occupational	No	Ref.	Ref.	Ref.	Ref.
		Yes	0.73	0.74	0.60	1.23
			(0.50–1.08)	(0.42–1.31)	(0.38–0.95)	(0.45–3.38)
Level 3	Alcoholism	No	Ref.	Ref.	Ref.	Ref.
		Yes	2.17	2.70	1.31	1.16
			(1.86–2.54)	(2.27–3.22)	(1.08–1.58)	(0.65–2.09)
	Mental disease	No	Ref.	Ref.	Ref.	Ref.
		Yes	1.22	0.65	0.75	0.33
			(0.94–1.58)	(0.45–0.93)	(0.53–1.04)	(0.08–1.42)
	Others comorbidities	No	Ref.	Ref.	Ref.	Ref.
		Yes	1.17	1.98	2.20	1.33
			(1.00–1.37)	(1.70–2.31)	(1.90–2.54)	(0.78–2.27)
	HIV/AIDS	No	Ref.	Ref.	Ref.	Ref.
		Yes	2.16	2.87	7.05	0.75
			(1.70–2.74)	(2.13–3.86)	(5.74–8.66)	(0.27–2.08)
Level 4	TB form	Pulmonary	Ref.	Ref.	Ref.	Ref.
		Extrapulmonary	0.93	0.88	1.51	0.59
			(0.76–1.14)	(0.68–1.14)	(1.27–1.78)	(0.13–2.61)
		Pulmonary +	0.93	2.49	1.95	1.36
		Extrapulmonary	(0.65–1.31)	(1.79–3.46)	(1.49–2.54)	---
	Initial smear	Negative	Ref.	Ref.	Ref.	Ref.
		Positive	0.85	0.79	0.64	2.38
			(0.74–0.97)	(0.66–0.95)	(0.56–0.73)	(1.25–4.53)
	Skin test	Negative	Ref.	Ref.	Ref.	Ref.
		Positive	0.75	0.37	0.53	0.57
			(0.57–0.99)	(0.24–0.57)	(0.41–0.68)	(0.19–1.71)
	Culture	Negative	Ref.	Ref.	Ref.	Ref.
		Positive	0.75	1.50	0.93	4.20
			(0.57–0.99)	(0.96–2.37)	(0.69–1.24)	(1.76–10.06)
	Hitopathologic	Not suggestive	Ref.	Ref.	Ref.	Ref.
		Suggestive of	0.53	0.73	0.53	5.72
		TB	(0.30–0.93)	(0.32–1.66)	(0.33–0.86)	---
		AFBpositive	0.70	1.12	0.83	0.23
			(0.40–1.23)	(0.48–2.59)	(0.51–1.36)	(0.06–0.94)
	2^nd^ month smear	Negative	Ref.	Ref.	Ref.	Ref.
		Positive	1.94	2.26	1.53	5.69
			(1.55–2.43)	(1.38–3.69)	(1.14–2.05)	(3.51–9.23)
Level 5	Treatment type	New case	Ref.	Ref.	Ref.	Ref.
		Relapse	1.75	1.41	1.28	9.60
			(1.45–2.10)	(1.08–1.85)	(1.05–1.57)	(6.07–15.14)
		Return after	5.41	2.02	1.34	17.13
		default	(4.47–6.54)	(1.44–2.85)	(0.97–1.84)	(9.58–30.63)
		Unknown	3.33	14.12	6.26	4.18
			(1.54–7.22)	(7.04–28.32)	(3.42–11.45)	---
		Transferred of	0.99	1.42	1.04	7.87
		treatment center	(0.78–1.26)	(1.07–1.88)	(0.83–1.30)	(4.74–13.07)
	DOT	No	Ref.	Ref.	Ref.	Ref.
		Yes	0.61	0.88	1.06	0.98
			(0.53–0.71)	(0.74–1.04)	(0.91–1.24)	(0.63–1.51)

MDR TB: multidrug resistant tuberculosis; TB: tuberculosis.

*Hierarchical levels: Level 1: Gender + Age + Skin color + Schooling. Level 2: Gender + Age + Skin color + Schooling + Area of residence + Institutionalization + Occupational. Level 3: Gender + Age + Skin color + Schooling + Area of residence + Institutionalization + Occupational + Alcoholism + Mental disease + HIV/AIDS + Other comorbidities. Level 4: Gender + Age + Skin color + Schooling + Area of residence + Institutionalization + Occupational + Alcoholism + Mental disease + HIV/AIDS + Other comorbidities + TB form + Initial smear + 2^nd^ month smear + Culture + Histopathologic + tuberculin skin test + X-ray. Level 5: Gender + Age + Skin color + Schooling + Area of residence + Institutionalization + Occupational + Alcoholism + Mental disease + HIV/AIDS + Other comorbidities + TB form + Initial smear + 2^nd^ month smear + Culture + Histopathologic + Tuberculin skin test + treatment type + directly observed therapy.

The odds of default was also higher among those in the age group 20–39 years (OR = 2.07, 95% CI 1.32–3.24), blacks (OR = 1.52, 95% CI 1.31–1.76) and browns (OR = 1.19, 95% CI 1.06–1.33).Those with alcoholism (OR = 2.17, 95% CI 1.86–2.54), HIV/AIDS (OR = 2.16, 95% CI 1.70–2.74) and others comorbidities (OR = 1.17, 95% CI 1.00–1.37), with positivity of smear at second month of treatment (OR = 1.94, 95% CI 1.55–2.43), TB relapse (OR = 1.75, 95% CI 1.45–2.10) and for those that return to treatment after prior default (OR = 5.41, 95% CI 4.47–6.54) and with unknown type of treatment (OR = 3.33, 95% CI 1.54–7.22) had higher odds of default.

The odds of death in TB-DM subjects was higher for those ≥60 years (OR = 2.74, 95% CI 1.74–4.29) compared to groups <20 years old; blacks (OR = 1.25, 95% CI 1.02–1.54) and browns (OR = 1.26, 95% CI 1.09–1.45) compared to whites; subjects institutionalized in shelter (OR = 2.69, 95% CI 1.07–6.77); with alcoholism (OR = 2.70, 95% CI 2.27–3.22); HIV/AIDS (OR = 2.87, 95% CI 2.13–3.86); others comorbidities (OR = 1.98, 95% CI 1.70–2.31), pulmonary plus extra pulmonary TB (OR = 2.49, 95% CI 1.79–3.46) and unknown type of treatment (OR = 14.12, 95% CI 7.04–28.32) compared to subjects treated as new cases.

On the other hand, subjects with 12–16 years of schooling were less likely to die compared to illiterate subjects (OR = 0.55, 95% CI 0.34–0.88), those with mental disease (OR = 0.65, 95% CI 0.45–0.93), positive initial smear (OR = 0.79, 95% CI 0.66–0.95) and positive tuberculin skin test (OR = 0.37, 95% CI 0.24–0.57).

Development of MDR TB was more related to relapse (OR = 9.60, 95% CI 6.07–15.14), return to treatment after default (OR = 17.13, 95% CI 9.58–30.63) and transfer of treatment center (OR = 7.87, 95% CI 4.74–13.07).

## Discussion

In spite of an overall decline inTB incidence in the country, diabetics among TB cases increased substantially and progressively during the 10-year period between 2001 and 2011. Still it is important to consider the possibility of under diagnosis since the DM definition was based on self-reported from the subjects at the TB diagnosis time. If the diabetes cases had not increased significantly, maybe the decrease in TB incidence would have been more important. The trend of increased convergence of TB and DM is perceived and discussed worldwide [17], [18] and this is already a topic in the World Health Organization agenda [19].

Some limitations should be mentioned. Missing data were not negligible and another limitation was that SINAN database does not include the diabetes type of the patients, features of comorbidities as HIV/AIDS, and culture and drug susceptibility test results at second month of treatment. In Brazil, *M. tuberculosis* culture is not routinely performed for all patients; culture and drug susceptibility tests are only recommended for special cases such as retreatment after failure, relapse, patients with suspected primary resistance and contacts of a resistant TB case [20]. In our data only 7.8% of the patients were tested by culture at the time of diagnosis.

The strengths of our study are its large sample size, the utilization of data based on an information system that has shown steady improvement in quality overtime [21], and the utilization of covariates stratified by socio-demographic and clinical characteristics. Furthermore this study arises to fill the need to know what differentiates the outcomes of treatment of diabetics identified in a previous publication that showed that those had poor outcomes [6]. Most studies generally examine dichotomous outcomes such as cure and default [22], whereas the use of polynomial analysis allowed inclusion of 5 outcomes present in the Notification System. By looking into the specific diabetes group we could be able to understand the differences in their outcomes. This type of analysis gives a more refined understanding of the different characteristics that may influence the outcomes as pointed by others [5].

Increased treatment outcome failures in male subjects have been previously reported in general population [23] and it is a worrying data since males are also more likely to acquire TB [2].

The mean age of the study population was 53±15 years. This age group is also at increased risk for type 2 diabetes [24]. Whereas cure was more likely in the younger age group (<20 years), the elderly patients (>60 years) had increased risk of death from TB; one possibility is that they might have had more advanced disease at the time of TB diagnosis. They also have, in general, a higher proportion of comorbidities, and a significantly higher mortality compared with the younger age groups [25]. Regarding default, the 20–39 years-old, an economically active population, showed an important percentage (12%), similarly to other studies that observed over 30% of the default in this age group [26].

The greater proportion of default and death among blacks and browns compared to whites followed previous findings where in general the non-white populations live in worst economic and social conditionsand consequently more barriers to successfully treatment [27]. Socioeconomic position is related with educational level that one has attained usually achieved in early adulthood, withthe occupation that one holds across adult life, with the income that one earns, and with the wealth that is accumulated over life. Since,TB time has deep social and economic roots, education is an important socioeconomic determinant, and its relationship with mortality was already described [28].

It is well established that TB is closely associated with poor living conditions, poverty, inadequate sanitary conditions, overcrowded households and small living quarters, and low socioeconomic status [23], [29]. Attention is needed in shelters where TB infection is common and sometimes untreated [30].

Default and death from TB were related with alcoholism even after control to socio-demographic characteristics. There are potential social pathways linking these 2 health problems: heavy alcohol use strongly influences both the incidence and the outcome of the disease, and it is linked to altered pharmacokinetics of medicines used in treatment of TB, social marginalization, higher rate of reinfection, higher rate of treatment defaults and development of drug-resistant forms of TB [31]. Based on our data we could not explain why patients with mental illness have better outcomes of TB treatment. This relationship demands more analysis in further studies.

The presence of others comorbidities was related to default and death from TB in our data. It is known that comorbidities such as HIV/AIDS, immunosuppressive therapy, silicosis, renal failure, cancer, corticosteroids use and smoking increase the risk of TB development and they also increase the risk of poor outcomes [29]. However, it is important to note that in a previous study with subjects with TB and chronic kidney disease in Brazil DM was not associated with undesirable TB treatment outcomes [32].

The risk of default and death also are significantly higher in HIV-infected patients with tuberculosis compared to HIV-uninfected patients [33]. The prevalence of serum positivity to HIV is increasing among diabetics worldwide [34] which makes it even more important the found association of HIV and AIDS with undesirable outcomes.

Development of MDR TB was found to be associated with relapse, return to treatment after default and transfer of treatment center. The process of resistance in tuberculosis is particularly serious for patients who have received prior treatment without success. In many of them, lesions advance by repeated reactivations and inadequate treatments, which can be a risk factor for mutant bacilli resistant to one or more drugs [35].

Disseminated disease was also associated with poor prognosis, as the combined pulmonary and extrapulmonary form, more associated with death than patient who presents only pulmonary TB or extrapulmonary TB forms [36]. Therefore, among subjects with DM and suspects of, health care professionals should be vigilant to be able to detect these forms of TB early enough in order to improve treatment outcomes. In addition, they should be looking for early signs of poor response to therapy. This can diminish the burden of treatment failure and death, which some studies have indicated to be more frequent in diabetic patients than in those without DM [37].

The cure rate of 80% found in this study is below the recommended target of least 85% set by the WHO [38]. Nevertheless, it is important to highlight that in this special population this cure rate was acceptable.Still, every effort should be made to improve this parameter specially highlighting older subjects, those with comorbidities and with a previous treatment of TB that presented poorest outcomes of TB treatment.

This study reported characteristics related to tuberculosis patients with diabetes, and the impact of the convergence of both epidemics in favouring poor individual outcomes, mainly death by TB and default. Besides diagnosis, TB control programs will need to expand efforts to focus on treatment follow-up to improve cure rates and achieve the goals of elimination of TB in the new global scenario of chronic diseases.
